# Supplementary material for: Choosing important health outcomes for comparative effectiveness research: 5th annual update to a systematic review of core outcome sets for research
Source: PLoS One. 2019 Dec 12;14(12):e0225980. doi: 10.1371/journal.pone.0225980 (PMC6907830; doi:10.1371/journal.pone.0225980)
Supplement: S2 Table — (DOCX) [file pone.0225980.s003.docx]

**S2 Table. Table of reports included in updated review (n=61)**

| **Study** | **Disease category** | **Disease name** |
| --- | --- | --- |
| Agha 2018 [1]**  Agha 2016 [2] | Cancer | Breast cancer |
| Allin 2018 [3]**  Ross 2016 [4] | Gastroenterology | Gastroschisis |
| Balakrishnan 2018 [5]* | Lungs and Airways | n/a |
| Beuscart 2018 [6]**  Beuscart 2017 [7] | Healthcare of older people | Polypharmacy |
| Callis Duffin 2018 [8]**  Perez-Chada 2018 [9] | Skin | Psoriasis |
| Chalmers 2018 [10]^  Howells 2017 [11] | Skin | Atopic eczema/dermatitis |
| Chiarotto 2018 [12]^  Chiarotto 2018 [13] | Orthopaedics & trauma | Low back pain |
| Coates 2018 [14]^  Holland 2018 [15] | Rheumatology | Psoriatic Arthritis |
| Danese 2018 [16]** | Gastroenterology | Inflammatory bowel disease |
| Dos Santos 2018 [17]** | Pregnancy and Childbirth | Induction of labour |
| Durnea 2018 [18]** | Gynaecology | Anterior-compartment vaginal prolapse |
| Fish 2018 [19]**  Fish 2018 [20] | Cancer | Anal cancer |
| Hall 2018 [21]**  Hall 2016 [22] | Ear, nose & throat | Tinnitus |
| Haywood 2018 [23]**  Whitehead 2015 [24] | Heart & Circulation | Cardiac arrest |
| Hoang 2017 [25]^  Singh 2017 [26]  Wall 2017 [27] | Rheumatology | Total joint replacement |
| Hopkins 2017 [28]**  Soni-Jaiswal 2017 [29] | Ear, nose & throat | Chronic rhinosinusitis (CRS) |
| Horbach 2018 [30]** | Heart & Circulation | Peripheral vascular malformations |
| Iorio 2018 [31]** | Blood disorders | Haemophilia |
| Kaiser 2018 [32]**  Deckert 2016 [33] | Anaesthesia & pain control | Chronic pain |
| McGrattan 2018 [34]** | Neurology | Dementia |
| Meher 2018 [35]** | Pregnancy & childbirth | Postpartum haemorrhage |
| Morgan 2017 [36]^ | Rheumatology | Juvenile idiopathic arthritis |
| Murugupillai 2018 [37]** | Neurology | Epilepsy |
| O’Donnell 2018 [38]**  O’Donnell 2018 [39] | Orthopaedics & trauma | Hip fracture |
| Olliaro 2018 [40]^ | Skin | Cutaneous leishmaniasis |
| Page 2018 [41]^ | Orthopaedics & trauma | Shoulder disorders |
| Paillaud 2018 [42]^ | Cancer | Cancer |
| Pergialiotis 2018 [43]** | Pregnancy & childbirth | Childbirth perineal trauma |
| Pukall 2017 [44]** | Gynaecology | Vulvodynia |
| Pushpanathan 2018 [45]** | Anaesthesia & pain control | Postoperative Pain |
| Radner 2018 [46]** | Rheumatology | Rheumatoid arthritis |
| Rankin 2018 [47]**  Nuha 2014 [48] | Healthcare of older people | Polypharmacy |
| Sahnan 2018 [49]** | Gastroenterolgy | Fistulising perianal Crohn’s disease |
| Sautenet 2018 [50]^  Tong 2018 [51] | Kidney disease | Kidney transplant |
| Singendonk 2018 [52]** | Gastroenterology | Infant Gastroesophageal Reflux Disease |
| Smith 2018 [53]** | Other | Multimorbidity |
| Spargo 2018 [54]**  McCullough 2015 [55] | Lungs & airways | Bronchiectasis |
| Sreih 2018 [56]^ | Rheumatology | Large-vessel vasculitis |
| Thorlacius 2018 [57]**  Thorlacius 2018 [58] | Skin | Hidradenitis suppurativa |
| van den Bussche 2018 [59]** | Skin | Incontinence-associated dermatitis |
| van Geel 2017 [60]^ | Skin | Vitiligo |
| Williams 2018 [61]^ | Anaesthesia & pain control | N/A |

*^ Linked to COS included in previous review*

** Considered outcomes while addressing wider clinical trial design issues*

*** Specifically considered outcome selection and measurement*

**References**

1. Agha RA, Pidgeon TE, Borrelli MR, et al. Validated Outcomes in the Grafting of Autologous Fat to the Breast: The VOGUE Study. Development of a Core Outcome Set for Research and Audit. Plast Reconstr Surg 2018;141(5):633e-8e.
2. Agha RA, Fowler AJ, Pidgeon TE, et al. The Need for Core Outcome Reporting in Autologous Fat Grafting for Breast Reconstruction. Ann Plas Surg 2016;77(5):506-12.
3. Allin BSR, Hall NJ, Ross AR, et al. Development of a gastroschisis core outcome set. Arch Dis Child: Fetal and Neonatal Edition 2018 Mar 14.
4. Ross AR, Hall NJ. Outcome reporting in randomized controlled trials and systematic reviews of gastroschisis treatment: a systematic review. J Pediatr Surg. 2016;51:1385-9.
5. Balakrishnan K, Sidell DR, Bauman NM, et al. Outcome measures for pediatric laryngotracheal reconstruction: International consensus statement. Laryngoscope 2018 Aug 27.
6. Beuscart JB, Knol W, Cullinan S, et al. International core outcome set for clinical trials of medication review in multi-morbid older patients with polypharmacy. BMC Med 2018;16(1):21.
7. Beuscart JB, Pont LG, Thevelin S, et al. A systematic review of the outcomes reported in trials of medication review in older patients: the need for a core outcome set. Br J Clin Pharmacol 2017;83(5):942-52.
8. Callis Duffin K, Merola JF, Christensen R, et al. Identifying a Core Domain Set to Assess Psoriasis in Clinical Trials. JAMA Dermatology 2018;154(10):1237-44.
9. Perez-Chada LM, Cohen JM, Gottlieb AB, et al. Achieving international consensus on the assessment of psoriatic arthritis in psoriasis clinical trials: an International Dermatology Outcome Measures (IDEOM) initiative. Arch Dermatol Res 2018;310(9):701-10.
10. Chalmers JR, Thomas KS, Apfelbacher C, et al. Report from the fifth international consensus meeting to harmonize core outcome measures for atopic eczema/dermatitis clinical trials (HOME initiative). Br J Dermatol 2018;178(5):e332-e41.
11. Howells LM, Chalmers JR, Cowdell F, et al. 'When it goes back to my normal I suppose': a qualitative study using online focus groups to explore perceptions of 'control' among people with eczema and parents of children with eczema in the UK. BMJ Open 2017;7(11):e017731.
12. Chiarotto A, Boers M, Deyo RA, et al. Core outcome measurement instruments for clinical trials in nonspecific low back pain. Pain 2018;159(3):481-95.
13. Chiarotto A, Ostelo RW, Boers M, et al. A systematic review highlights the need to investigate the content validity of patient-reported outcome measures for physical functioning in patients with low back pain. J Clin Epidemiol 2018;95:73-93.
14. Coates LC, FitzGerald O, Merola JF, et al. Group for Research and Assessment of Psoriasis and Psoriatic Arthritis/Outcome Measures in Rheumatology Consensus-Based Recommendations and Research Agenda for Use of Composite Measures and Treatment Targets in Psoriatic Arthritis. Arthritis Rheumatol 2018;70(3):345-55.
15. Holland R, Tillett W, Ogdie A, et al. Content and Face Validity and Feasibility of 5 Candidate Instruments for Psoriatic Arthritis Randomized Controlled Trials: The PsA OMERACT Core Set Workshop at the GRAPPA 2017 Annual Meeting. J Rheumatol Suppl 2018;94:17-25.
16. Danese S, Bonovas S, Lopez A, et al. Identification of Endpoints for Development of Antifibrosis Drugs for Treatment of Crohn's Disease. Gastroenterology 2018;155(1):76-87.
17. Dos Santos F, Drymiotou S, Antequera Martin A, et al. Development of a core outcome set for trials on induction of labour: an international multistakeholder Delphi study. BJOG 2018;125(13):1673-80.
18. Durnea CM, Pergialiotis V, Duffy JMN, et al. A systematic review of outcome and outcome-measure reporting in randomised trials evaluating surgical interventions for anterior-compartment vaginal prolapse: a call to action to develop a core outcome set. Int Urogynecol J 2018;29(12):1727-45.
19. Fish R, Sanders C, Adams R, et al. A core outcome set for clinical trials of chemoradiotherapy interventions for anal cancer (CORMAC): a patient and health-care professional consensus. Lancet Gastroenterol Hepatol 2018;3(12):865-73.
20. Fish R, Sanders C, Ryan N, et al. Systematic review of outcome measures following chemoradiotherapy for the treatment of anal cancer (CORMAC). Colorectal Dis 2018;20(5):371-82.
21. Hall DA, Smith H, Hibbert A, et al. The COMiT’ID Study: Developing Core Outcome Domains Sets for Clinical Trials of Sound-, Psychology-, and Pharmacology-Based Interventions for Chronic Subjective Tinnitus in Adults. Trends Hear 2018;22.
22. Hall DA, Haider H, Szczepek AJ, et al. Systematic review of outcome domains and instruments used in clinical trials of tinnitus treatments in adults. Trials 2016;17(1):270.
23. Haywood K, Whitehead L, Nadkarni VM, et al. COSCA (Core Outcome Set for Cardiac Arrest) in Adults: An Advisory Statement From the International Liaison Committee on Resuscitation. Resuscitation 2018;127:147-63.
24. Whitehead L, Perkins GD, Clarey A, et al. A systematic review of the outcomes reported in cardiac arrest clinical trials: the need for a core outcome set. Resuscitation 2015;88:150-7.
25. Hoang A, Goodman SM, Navarro-Millan IY, et al. Patients and surgeons provide endorsement of core domains for total joint replacement clinical trials. Arthritis Res Ther 2017;19(1):267.
26. Singh JA, Dowsey MM, Dohm M, et al. Achieving Consensus on Total Joint Replacement Trial Outcome Reporting Using the OMERACT Filter: Endorsement of the Final Core Domain Set for Total Hip and Total Knee Replacement Trials for Endstage Arthritis. J Rheumat 2017;44(11):1723-6.
27. Wall PDH, Richards BL, Sprowson A, et al. Do outcomes reported in randomised controlled trials of joint replacement surgery fulfil the OMERACT 2.0 Filter? A review of the 2008 and 2013 literature. Syst Rev 2017;6(1):106.
28. Hopkins C, Hettige R, Soni-Jaiswal A, et al. CHronic rhinosinusitis outcome MEasures (CHROME) – developing a core outcome set for trials of interventions in chronic rhinosinusitis. Rhinology 2018;56(1):22-32.
29. Soni-Jaiswal A, Lakhani R, Hopkins C. Developing a core outcome set for chronic rhinosinusitis: a systematic review of outcomes utilised in the current literature. Trials 2017;18(1):320.
30. Horbach SER, van der Horst CMAM, Blei F, et al. Development of an international core outcome set for peripheral vascular malformations: the OVAMA project. Br J Dermatol 2018;178(2):473-81.
31. Iorio A, Skinner MW, Clearfield E, et al. Core outcome set for gene therapy in haemophilia: Results of the coreHEM multistakeholder project. Haemophilia 2018;24(4):e167-e72.
32. Kaiser U, Kopkow C, Deckert S, et al. Developing a core outcome domain set to assessing effectiveness of interdisciplinary multimodal pain therapy: The VAPAIN consensus statement on core outcome domains. Pain 2018;159(4):673-83.
33. Deckert S, Kaiser U, Kopkow C, et al. A systematic review of the outcomes reported in multimodal pain therapy for chronic pain. Eur J Pain 2016;20:51-63.
34. McGrattan M, Barry HE, Ryan C, et al. The development of a Core Outcome Set for medicines management interventions for people with dementia in primary care. Age Ageing 2018 Nov 5.
35. Meher S, Cuthbert A, Kirkham JJ, et al. Core outcome sets for prevention and treatment of postpartum haemorrhage: an international Delphi consensus study. BJOG 2018 Jul 29.
36. Morgan EM, Riebschleger MP, Horonjeff J, et al. Evidence for Updating the Core Domain Set of Outcome Measures for Juvenile Idiopathic Arthritis: Report from a Special Interest Group at OMERACT 2016. J Rheumatol 2017;44(12):1884-8.
37. Murugupillai R, Ranganathan SS, Wanigasinghe J, et al. Development of outcome criteria to measure effectiveness of antiepileptic therapy in children. Epilepsy Behav 2018;80:56-60.
38. O’Donnell CM, McLoughlin L, Patterson CC, et al. Perioperative outcomes in the context of mode of anaesthesia for patients undergoing hip fracture surgery: a systematic review and meta-analysis. Br J Anaesth 2018;120:37e50
39. O'Donnell CM, Black N, McCourt KC, et al. Development of a Core Outcome Set for studies evaluating the effects of anaesthesia on perioperative morbidity and mortality following hip fracture surgery. Br J Anaesth 2018 Oct 3.
40. Olliaro P, Grogl M, Boni M, et al. Harmonized clinical trial methodologies for localized cutaneous leishmaniasis and potential for extensive network with capacities for clinical evaluation. PLoS Negl Trop Dis 2018;12(1):e0006141.
41. Page MJ, Huang H, Verhagen AP, et al. Outcome Reporting in Randomized Trials for Shoulder Disorders: Literature Review to Inform the Development of a Core Outcome Set. Arthritis Care Res 2018;70(2):252-9.
42. Paillaud E, Soubeyran P, Caillet P, et al. Multidisciplinary development of the Geriatric Core Dataset for clinical research in older patients with cancer: A French initiative with international survey. Eur J Cancer 2018;103:61-8.
43. Pergialiotis V, Durnea C, Elfituri A, et al. Do we need a core outcome set for childbirth perineal trauma research? A systematic review of outcome reporting in randomised trials evaluating the management of childbirth trauma. BJOG 2018;125(12):1522-31.
44. Pukall CF, Bergeron S, Brown C, et al. Recommendations for Self-Report Outcome Measures in Vulvodynia Clinical Trials. Clin J Pain 2017;33(8):756-65.
45. Pushpanathan E, Setty T, Carvalho B, et al. A Systematic Review of Postoperative Pain Outcome Measurements Utilised in Regional Anesthesia Randomized Controlled Trials. Anesthesiol Res Pract 2018;2018.
46. Radner H, Chatzidionysiou K, Nikiphorou E, et al. 2017 EULAR recommendations for a core data set to support observational research and clinical care in rheumatoid arthritis. Ann Rheum Dis 2018;77(4):476-9.
47. Rankin A, Cadogan CA, Ryan C, et al. Core Outcome Set for Trials Aimed at Improving the Appropriateness of Polypharmacy in Older People in Primary Care. J Am Geriatr Soc 2018;66(6):1206-12.
48. Nuha A, Robin K, Ailsa L, et al. The anal fistula journal: effects on patients’ life experiences. In: Gastroenterology 2014;146:S-207-–0.
49. Sahnan K, Tozer PJ, Adegbola SO, et al. Developing a core outcome set for fistulising perianal Crohn's disease. Gut 2018 Feb 3.
50. Sautenet B, Tong A, Chapman JR, et al. Range and Consistency of Outcomes Reported in Randomized Trials Conducted in Kidney Transplant Recipients: A Systematic Review. Transplantation 2018;102(12):2065-71.
51. Tong A, Sautenet B, Poggio ED, et al. Establishing a Core Outcome Measure for Graft Health: A Standardized Outcomes in Nephrology-Kidney Transplantation (SONG-Tx) Consensus Workshop Report. Transplantation 2018;102(8):1358-66.
52. Singendonk MMJ, Rexwinkel R, Steutel NF, et al. Development of A Core Outcome Set For Infant Gastroesophageal Reflux Disease. J Pediatr Gastroenterol Nutr 2018 Dec 27.
53. Smith SM, Wallace E, Salisbury C, et al. A Core Outcome Set for Multimorbidity Research (COSmm). Ann Fam Med 2018;16(2):132-8.
54. Spargo M, Ryan C, Downey D, et al. Development of a core outcome set for trials investigating the long-term management of bronchiectasis. Chronic Respiratory Disease 2018 Oct 3.
55. McCullough AR, Ryan C, O’Neill B, et al. Defining the content and delivery of an intervention to Change AdhereNce to treatment in BonchiEctasis (CAN-BE): a qualitative approach incorporating the theoretical domains framework, behavioural change techniques and stakeholder expert panels. BMC Health Serv Res 2015;15:342-352.
56. Sreih AG, Alibaz-Oner F, Easley E, et al. Health-related outcomes of importance to patients with Takayasu's arteritis. Clin Exp Rheumatol 2018;36 Suppl 111(2):51-7.
57. Thorlacius L, Ingram JR, Villumsen B, et al. A core domain set for hidradenitis suppurativa trial outcomes: an international Delphi process. Br J Dermatol 2018;179(3):642-50.
58. Thorlacius L, Garg A, Ingram JR et al. Towards global consensus on core outcomes for hidradenitis suppurativa research: an update from the HISTORIC consensus meetings I and II. Br J Dermatol 2018; 178:715–21.
59. Van den Bussche K, Kottner J, Beele H, et al. Core outcome domains in incontinence-associated dermatitis research. J Adv Nurs 2018;74(7):1605-17.
60. van Geel N, Boniface K, Seneschal J, et al. Meeting report: Vitiligo Global Issues Consensus Conference Workshop "Outcome measurement instruments" and Vitiligo International Symposium, Rome, Nov 30-Dec 3rd. Pigment Cell Melanoma Res 2017;30(4):436-43.
61. Williams MR, Nayshtut M, Hoefnagel A, et al. Efficacy Outcome Measures for Pediatric Procedural Sedation Clinical Trials: An ACTTION Systematic Review. Anesth Analg 2018;126(3):956-67.
