# Supplementary material for: Choosing important health outcomes for comparative effectiveness research: 5th annual update to a systematic review of core outcome sets for research
Source: PLoS One. 2019 Dec 12;14(12):e0225980. doi: 10.1371/journal.pone.0225980 (PMC6907830; doi:10.1371/journal.pone.0225980)
Supplement: S3 Table — (DOCX) [file pone.0225980.s004.docx]

**S3 Table.** COS minimum standards: assessment by study (n=30)

| **Key ✓** = standard met **O**  = unclear whether standard is met  **X** = Standard not met | | | | | | | | | | | | |
| --- | --- | --- | --- | --- | --- | --- | --- | --- | --- | --- | --- | --- |
|  | **Scope** | | | | **Stakeholders** | | | **Consensus Process** | | | | |
| **Standard number** | **1** | **2** | **3** | **4** | 5 | **6** | **7** | **8** | **9a** | **9b** | **10** | **11** |
| Agha et al (2018) [1] | ✓ | ✓ | ✓ | ✓ | ✓ | ✓ | x | x | ✓ | ✓ | ✓ | o |
| Allin et al (2019) [2] | ✓ | ✓ | ✓ | ✓ | ✓ | ✓ | ✓ | x | ✓ | ✓ | ✓ | ✓ |
| Balakrishnan et al (2019) [3] | ✓ | ✓ | ✓ | ✓ | ✓ | ✓ | x | x | ✓ | ✓ | O | O |
| Beuscart et al (2018) [4] | ✓ | ✓ | ✓ | ✓ | ✓ | ✓ | ✓ | ✓ | ✓ | ✓ | ✓ | ✓ |
| Callis Duffin et al (2018) [5] | ✓ | ✓ | ✓ | ✓ | ✓ | ✓ | ✓ | ✓ | O | O | x | ✓ |
| Danese et al (2018) [6] | ✓ | ✓ | ✓ | ✓ | x | ✓ | x | x | O | O | O | O |
| Dos Santos et al (2018) [7] | ✓ | ✓ | ✓ | ✓ | ✓ | ✓ | ✓ | O | ✓ | ✓ | O | ✓ |
| Durnea et al (2018) [8] | ✓ | ✓ | ✓ | ✓ | x | x | x | x | x | x | x | x |
| Fish et al (2018) [9] | ✓ | ✓ | ✓ | ✓ | ✓ | ✓ | ✓ | ✓ | ✓ | ✓ | ✓ | ✓ |
| Hall et al (2018) [10] | ✓ | ✓ | ✓ | ✓ | ✓ | ✓ | ✓ | ✓ | ✓ | ✓ | ✓ | ✓ |
| Haywood et al (2018) [11] | ✓ | ✓ | ✓ | ✓ | ✓ | ✓ | ✓ | ✓ | O | O | O | O |
| Hopkins et al (2018) [12] | ✓ | ✓ | ✓ | ✓ | ✓ | ✓ | ✓ | ✓ | ✓ | ✓ | ✓ | O |
| Horbach et al (2018) [13] | ✓ | ✓ | ✓ | ✓ | ✓ | ✓ | ✓ | ✓ | O | O | O | ✓ |
| Iorio et al (2018) [14] | ✓ | ✓ | ✓ | ✓ | ✓ | ✓ | ✓ | O | O | O | O | O |
| Kaiser et al (2018) [15] | ✓ | ✓ | ✓ | ✓ | ✓ | ✓ | ✓ | ✓ | ✓ | ✓ | O | O |
| McGrattan et al (2018) [16] | ✓ | ✓ | ✓ | ✓ | ✓ | ✓ | ✓ | ✓ | ✓ | ✓ | ✓ | O |
| Meher et al (2019) [17] | ✓ | ✓ | ✓ | ✓ | ✓ | ✓ | ✓ | x | ✓ | ✓ | ✓ | ✓ |
| Murugupillai et al (2018) [18] | ✓ | ✓ | ✓ | ✓ | ✓ | ✓ | ✓ | ✓ | O | O | O | O |
| O'Donnell et al (2019) [19] | ✓ | ✓ | ✓ | ✓ | ✓ | ✓ | ✓ | x | ✓ | ✓ | ✓ | O |
| Pergialiotis et al (2018) [20] | ✓ | ✓ | ✓ | ✓ | x | x | X | x | x | x | x | x |
| Pukall et al (2017) [21] | ✓ | ✓ | ✓ | ✓ | ✓* | ✓* | x* | x* | O | O | O | O |
| Pushpanathan et al (2018) [22] | ✓ | ✓ | ✓ | ✓ | x | x | x | x | x | x | x | x |
| Radner et al (2018) [23] | ✓ | ✓ | ✓ | ✓ | ✓ | ✓ | ✓ | x | O | O | O | O |
| Rankin et al (2018) [24] | ✓ | ✓ | ✓ | ✓ | ✓ | ✓ | ✓ | ✓ | ✓ | ✓ | ✓ | ✓ |
| Sahnan et al (2019) [25] | ✓ | ✓ | ✓ | ✓ | x | ✓ | ✓ | ✓ | ✓ | ✓ | ✓ | ✓ |
| Singendonk et al (2018) [26] | ✓ | ✓ | ✓ | ✓ | x | ✓ | ✓ | ✓ | O | O | x | O |
| Smith et al (2018) [27] | ✓ | ✓ | ✓ | ✓ | ✓ | ✓ | ✓ | x | ✓ | ✓ | ✓ | ✓ |
| Spargo et al (2018) [28] | ✓ | ✓ | ✓ | ✓ | ✓ | ✓ | ✓ | ✓ | ✓ | ✓ | ✓ | ✓ |
| Thorlacius et al (2018) [29] | ✓ | ✓ | ✓ | ✓ | ✓ | ✓ | ✓ | ✓ | ✓ | ✓ | ✓ | ✓ |
| Van den Bussche et al (2018) [30] | ✓ | ✓ | ✓ | ✓ | ✓ | ✓ | ✓ | ✓ | ✓ | ✓ | O | O |

*From author

**References**

1. Agha, R.A., et al., *Validated Outcomes in the Grafting of Autologous Fat to the Breast: The VOGUE Study. Development of a Core Outcome Set for Research and Audit.* Plastic & Reconstructive Surgery, 2018. **141**(5): p. 633e-638e.

2. Allin, B.S.R., et al., *Development of a gastroschisis core outcome set.* Archives of Disease in Childhood: Fetal and Neonatal Edition, 2019. **104**(1): p. F76-F82.

3. Balakrishnan, K., et al., *Outcome measures for pediatric laryngotracheal reconstruction: International consensus statement.* Laryngoscope, 2019. **129**(1): p. 244-255.

4. Beuscart, J.B., et al., *International core outcome set for clinical trials of medication review in multi-morbid older patients with polypharmacy.* BMC Medicine, 2018. **16**(1): p. 21.

5. Callis Duffin, K., et al., *Identifying a Core Domain Set to Assess Psoriasis in Clinical Trials.* JAMA Dermatology, 2018. **154**(10): p. 1237-1244.

6. Danese, S., et al., *Identification of Endpoints for Development of Antifibrosis Drugs for Treatment of Crohn's Disease.* Gastroenterology, 2018. **155**(1): p. 76-87.

7. Dos Santos, F., et al., *Development of a core outcome set for trials on induction of labour: an international multistakeholder Delphi study.* BJOG: An International Journal of Obstetrics and Gynaecology, 2018. **125**(13): p. 1673-1680.

8. Durnea, C.M., et al., *A systematic review of outcome and outcome-measure reporting in randomised trials evaluating surgical interventions for anterior-compartment vaginal prolapse: a call to action to develop a core outcome set.* International Urogynecology Journal, 2018. **29**(12): p. 1727-1745.

9. Fish, R., et al., *A core outcome set for clinical trials of chemoradiotherapy interventions for anal cancer (CORMAC): a patient and health-care professional consensus.* The Lancet Gastroenterology and Hepatology, 2018. **3**(12): p. 865-873.

10. Hall, D.A., et al., *The COMiT’ID Study: Developing Core Outcome Domains Sets for Clinical Trials of Sound-, Psychology-, and Pharmacology-Based Interventions for Chronic Subjective Tinnitus in Adults.* Trends in Hearing, 2018. **22**.

11. Haywood, K., et al., *COSCA (Core Outcome Set for Cardiac Arrest) in Adults: An Advisory Statement From the International Liaison Committee on Resuscitation.* Resuscitation, 2018. **127**: p. 147-163.

12. Hopkins, C., et al., *CHronic rhinosinusitis outcome MEasures (CHROME) – developing a core outcome set for trials of interventions in chronic rhinosinusitis.* Rhinology, 2018. **56**(1): p. 22-32.

13. Horbach, S.E.R., et al., *Development of an international core outcome set for peripheral vascular malformations: the OVAMA project.* British Journal of Dermatology, 2018. **178**(2): p. 473-481.

14. Iorio, A., et al., *Core outcome set for gene therapy in haemophilia: Results of the coreHEM multistakeholder project.* Haemophilia, 2018. **24**(4): p. e167-e172.

15. Kaiser, U., et al., *Developing a core outcome domain set to assessing effectiveness of interdisciplinary multimodal pain therapy: The VAPAIN consensus statement on core outcome domains.* Pain, 2018. **159**(4): p. 673-683.

16. McGrattan, M., et al., *The development of a Core Outcome Set for medicines management interventions for people with dementia in primary care.* Age Ageing, 2019. **48**(2): p. 260-266.

17. Meher, S., et al., *Core outcome sets for prevention and treatment of postpartum haemorrhage: an international Delphi consensus study.* BJOG: An International Journal of Obstetrics and Gynaecology, 2019. **126**(1): p. 83-93.

18. Murugupillai, R., et al., *Development of outcome criteria to measure effectiveness of antiepileptic therapy in children.* Epilepsy and Behavior, 2018. **80**: p. 56-60.

19. O'Donnell, C.M., et al., *Development of a Core Outcome Set for studies evaluating the effects of anaesthesia on perioperative morbidity and mortality following hip fracture surgery.* British Journal of Anaesthesia, 2019. **122**(1): p. 120-130.

20. Pergialiotis, V., et al., *Do we need a core outcome set for childbirth perineal trauma research? A systematic review of outcome reporting in randomised trials evaluating the management of childbirth trauma.* BJOG: An International Journal of Obstetrics and Gynaecology, 2018. **125**(12): p. 1522-1531.

21. Pukall, C.F., et al., *Recommendations for Self-Report Outcome Measures in Vulvodynia Clinical Trials.* Clinical Journal of Pain, 2017. **33**(8): p. 756-765.

22. Pushpanathan, E., et al., *A Systematic Review of Postoperative Pain Outcome Measurements Utilised in Regional Anesthesia Randomized Controlled Trials.* Anesthesiology Research and Practice, 2018. **2018**.

23. Radner, H., et al., *2017 EULAR recommendations for a core data set to support observational research and clinical care in rheumatoid arthritis.* Annals of the Rheumatic Diseases, 2018. **77**(4): p. 476-479.

24. Rankin, A., et al., *Core Outcome Set for Trials Aimed at Improving the Appropriateness of Polypharmacy in Older People in Primary Care.* Journal of the American Geriatrics Society, 2018. **66**(6): p. 1206-1212.

25. Sahnan, K., et al., *Developing a core outcome set for fistulising perianal Crohn's disease.* Gut, 2019. **68**(2): p. 226-238.

26. Singendonk, M.M.J., et al., *Development of a Core Outcome Set for Infant Gastroesophageal Reflux Disease.* J Pediatr Gastroenterol Nutr, 2019. **68**(5): p. 655-661.

27. Smith, S.M., et al., *A Core Outcome Set for Multimorbidity Research (COSmm).* Annals of Family Medicine, 2018. **16**(2): p. 132-138.

28. Spargo, M., et al., *Development of a core outcome set for trials investigating the long-term management of bronchiectasis.* Chronic Respiratory Disease, 2018. **16**.

29. Thorlacius, L., et al., *A core domain set for hidradenitis suppurativa trial outcomes: an international Delphi process.* British Journal of Dermatology, 2018. **179**(3): p. 642-650.

30. Van den Bussche, K., et al., *Core outcome domains in incontinence-associated dermatitis research.* Journal of Advanced Nursing, 2018. **74**(7): p. 1605-1617.
