# Supplementary material for: Choosing important health outcomes for comparative effectiveness research: 5th annual update to a systematic review of core outcome sets for research
Source: PLoS One. 2019 Dec 12;14(12):e0225980. doi: 10.1371/journal.pone.0225980 (PMC6907830; doi:10.1371/journal.pone.0225980)
Supplement: Attachment — Submitted filename: Response to reviewer comments_Final.docx [file pone.0225980.s005.docx]

24th October 2019

Dear Editor

PONE-D-19-22167

The use of automated article ranking in the fifth annual update to a systematic review of core outcome sets for research

PLOS ONE

Many thanks to you and the reviewers for your constructive comments on our manuscript. It has been valuable to revisit the manuscript. In our response to reviewers we have outlined how each of the comments has been addressed in the revised manuscript, which we hope is now ready for publication.

Furthermore, we have updated the title to be more in keeping with the previous updates to this systematic review:

Choosing important health outcomes for comparative effectiveness research: 5th annual update to a systematic review of core outcome sets for research

As requested, I have included the updated Competing Interests Statement:

EG and PW are members of the COMET Management Group. All other authors had no competing interests. This does not alter our adherence to PLOS ONE policies on sharing data and materials.

Please include the updated funding statement:

Professor Williamson is a National Institute for Health Research (NIHR) Senior Investigator. The views expressed in this article are those of the author(s) and not necessarily those of the NIHR, or the Department of Health and Social Care.

Many thanks again for considering our work for PLOS ONE.

Yours sincerely

Dr Elizabeth Gargon

University of Liverpool

On behalf of

Sarah L. Gorst and Paula R. Williamson

**Journal requirements:**

1. Please ensure that your manuscript meets PLOS ONE's style requirements, including those for file naming. The PLOS ONE style templates can be found at
<http://www.journals.plos.org/plosone/s/file?id=wjVg/PLOSOne_formatting_sample_main_body.pdf> and <http://www.journals.plos.org/plosone/s/file?id=ba62/PLOSOne_formatting_sample_title_authors_affiliations.pdf>

Response: We have checked that the manuscript matches style requirements and made any necessary changes.

2. Thank you for stating the following in the Competing Interests section:

'EG and PW are members of the COMET Management Group. SG has no competing interests.'

Please confirm that this does not alter your adherence to all PLOS ONE policies on sharing data and materials, by including the following statement: "This does not alter our adherence to  PLOS ONE policies on sharing data and materials.” (as detailed online in our guide for authors <http://journals.plos.org/plosone/s/competing-interests>).

* Please include your updated Competing Interests statement in your cover letter; we will change the online submission form on your behalf.

 Response: We have added this sentence into the response letter.

**Reviewers' comments:**


**Reviewer #1:**

**Introduction : (minor comments)**
Line 62 : the first sentence sounds like if « saving money » was the final goal of COS research. Is it ? Don’t you think that choosing meaningful outcomes could also improve patient’s condition ?

Response: We agree that the inclusion of patient-important outcomes is one of the main benefits/features of COS, and have added an opening sentence into the introduction about the importance of outcomes being relevant to patients.

Line 73 : is this « awareness » only a COS developer awareness or also an awareness of COS users (trialists ? )

Response: There is an increased awareness in both of these groups. We have added in a sentence, and a reference to a survey of COMET database users, that highlights this.

Line 75 : could you please cite any recent « guidance and standards » such as the COMET guidelines or more recently the revised OMERACT handbook. Do you think that these guidelines are the only method for COS development or is there any room for other methods ?

Response: References to the COMET Handbook and standards have been added in, as well as a sentence to say that “As methodological research in this area is still in its infancy, COMET guidance and standards will be updated in the future to reflect new evidence as it becomes available.”

Line 86 : are you aware of any initiative to implement MeSH with headings for COS ? It would be very helpful

Response: Thanks for this comment. This is an ongoing discussion within the COMET Management Group. The introduction of a COS MeSH term in MEDLINE and other bibliographic databases for identifying COS papers would most definitely be very helpful. However, the inclusion of a new MeSH term in itself might be a challenge. MeSH is the National Library of Medicine’s controlled vocabulary thesaurus, who state that before a new descriptor is introduced, consideration must be given to how much is published about that topic; if little is published the Library see little purpose or advantage in creating a new descriptor in a vocabulary which has to encompass the subject content of the entire published literature. Although COS development is a growing area of research, it is relatively niche when compared to the broad nature of most medical subject headings. It is therefore probable that the proposal of a COS heading is unrealistic at this time. In the absence of a COS MeSH term, standards for reporting and development, as highlighted in the introduction to this paper, help with the searching and identification of COS studies. The use of standardised terms, for example ‘core outcome set’ in the title and abstract make it much easier to identify appropriate COS development papers.

Line 98 : using an automated screening approach is not an aim but a method. You already proved it worked in a previous paper cited ref 11.

Response: We have edited this to ‘test’ instead of use.

Line 99 : using the COS-STAD is not an aim but a method also. The aim is to check if the included COS were developed with reasonable methods (as formulated by Kirkham 2017)

Response: Edit made.

**METHODS : (major comment)**

Line 145 : Two comments to express that I doubt that only one researcher without medical background can assess the development of COSs for a large diversity of disease. The quality of assessment may be discussable.
Only one researcher assessed the included COS against COS Stad criteria. A double assessment of two researchers, one with an expertise in the given disease would be more transparent and relevant.

Response: Thank you for this comment. Our assessment was made on whether there was evidence in the paper(s) that the specific COS-STAD criteria were considered. This assessment does not require clinical expertise. However an assessment of the applicability or usefulness of a COS in a particular scenario would need to be evaluated *by the user* for the *intended purpose of use.* In this instance, it is likely that relevant clinical knowledge and understanding would be vital. We have added a comment about this into the discussion (see 2^nd^ paragraph lines 401-404 in revised manuscript).

Moreover, the table 2 of the reference 8 has been made for cancer while supplementary data 3 show that cancer COSs were a minority. Did you adapt the criteria for each disease, for instance criteria 3 ? I doubt that it could be done without the advice of an expert of the disease.

Response: Table 2 of the previous assessment of COS against COS-STAD was developed using cancer COS as an example, but not specifically for cancer COS. It was intended to facilitate users being able to compare a published COS to the standards. The paper states that consideration should be given to the specific disease area being considered (criteria 3), and that users need to use their own judgement regarding the scope for the purpose of use they require. Table 2 of the previous paper was only used to provide some guidance on *how* to compare the COS to the standards when needed, but the standards themselves were used for the assessment and the standards (COS-STAD) were developed to be applicable across diseases.

We have edited this sentence to ‘remove the word ‘cancer’ to avoid any confusion.

**DISCUSSION (minor comments)**
Line 369 : It could be useful to have an online access to the assessment of each published tools on the COMET website for instance ie a table with the disease, the COS, and the COS-STAD assessment.

Response: Thanks for this comment. We are currently considering this. While we recognise the potential merit of this, there is a need for individual consideration by the user of a COS for the specific intended purpose, therefore we have concerns that by including our assessment it could detract from people making their own critical assessment. However, we recognise this could be useful to some users, and are therefore considering it for our upcoming update to the COMET website and database, coming in 2020.

**Reviewer #2:**

The title of the paper is misleading. No information about the algorithm is given in the paper. I am sorry, but without this information it does not make sense for me to review this paper. I strongly recommend to change the title of the paper.

Response: We have updated the title to be more in keeping with the previous updates to this systematic review:

Choosing important health outcomes for comparative effectiveness research: 5th annual update to a systematic review of core outcome sets for research

**Reviewer #3:**

The following manuscript reports an important and updated systematic review of COS published in 2018.
This review is an update of a yearly published systematic review, methods are well know and described.
Results are clear and comprehensive.

Response: Thank you for these comments.

I only have a couple of comments on a single point:
- the authors aimed at assessing the use of an automated screening approach prospectively. Could the authors elaborate on this in the methods section?

Response: Thank you for this comment. We have added in to the methods section and the results section the estimated time saved by using this method.

- in the discussion, p21, lignes 364 and 366 : is there two different automated processes? from my understanding of the methods reported in the manuscript the authors used an automated screening method to rank the citations also reported as an automated ranking process.

Response: Your understanding is correct. We have edited this sentence to make this clearer.
